# Verification of IMRT dose calculations using AAA and PBC algorithms in dose buildup regions

**DOI:** 10.1120/jacmp.v11i4.3351

**Published:** 2010-08-26

**Authors:** Arun S. Oinam, Lakhwant Singh

**Affiliations:** ^1^ Department of Radiotherapy Post Graduate Institute of Medical Education and Research Chandigarh 160012 India; ^2^ Department of Physics Guru Nanak Dev University Amritsar 143005 India

**Keywords:** anisotropic analytical algorithm (AAA), pencil beam convolution algorithm (PBC), high‐dose buildup region, low‐dose buildup region, TLD, dose calculation, IMRT

## Abstract

The purpose of this comparative study was to test the accuracy of anisotropic analytical algorithm (AAA) and pencil beam convolution (PBC) algorithms of Eclipse treatment planning system (TPS) for dose calculations in the low‐ and high‐dose buildup regions. AAA and PBC algorithms were used to create two intensity‐modulated radiotherapy (IMRT) plans of the same optimal fluence generated from a clinically simulated oropharynx case in an in‐house fabricated head and neck phantom. The TPS computed buildup doses were compared with the corresponding measured doses in the phantom using thermoluminescence dosimeters (TLD 100). Analysis of dose distribution calculated using PBC and AAA shows an increase in gamma value in the dose buildup region indicating large dose deviation. For the surface areas of 1, 50 and $100\;{\text{cm}}^{2}$, PBC overestimates doses as compared to AAA calculated value in the range of 1.34%–3.62% at 0.6 cm depth, 1.74%–2.96% at 0.4 cm depth, and 1.96%–4.06% at 0.2 cm depth, respectively. In high‐dose buildup region, AAA calculated doses were lower by an average of ‐7.56%(SD=4.73%), while PBC was overestimated by 3.75%(SD=5.70%) as compared to TLD measured doses at 0.2 cm depth. However, at 0.4 and 0.6 cm depth, PBC overestimated TLD measured doses by 5.84%(SD=4.38%) and 2.40%(SD=4.63%), respectively, while AAA underestimated the TLD measured doses by ‐0.82%(SD=4.24%) and ‐1.10%(SD=4.14%) at the same respective depth. In low‐dose buildup region, both AAA and PBC overestimated the TLD measured doses at all depths except ‐2.05%(SD=10.21%) by AAA at 0.2 cm depth. The differences between AAA and PBC at all depths were statistically significant (p<0.05) in high‐dose buildup region, whereas it is not statistically significant in low‐dose buildup region. In conclusion, AAA calculated the dose more accurately than PBC in clinically important high‐dose buildup region at 0.4 cm and 0.6 cm depths. The use of an orfit cast increases the dose buildup effect, and this buildup effect decreases with depth.

PACS number: 87.53.Bn

## I. INTRODUCTION

Accurate calculation of dose distribution in the buildup region still remains a challenge to most of the commercially available photon dose calculation algorithms. This is primarily due to difficulties in modeling the contribution of doses from contaminated electrons originated from flattening filter, collimator assembly and, to a lesser extent, secondary scatter photons from the accelerator head.^(^ ^1^ ^–^ ^5^ ^)^ The problem is further complicated by oblique incidence of the beam and the use of multileaf collimator (MLC) for beam intensity modulation in treatment techniques like intensity‐modulated radiotherapy (IMRT).^(^ ^6^ ^)^ Several authors have reported measurement of skin dose on patient and buildup dose on phantom from different treatment techniques.^(^ ^6^ ^–^ ^7^ ^,^ ^8^ ^)^ While one study reported increase in skin dose of patients undergoing IMRT treatment,^(^ ^9^ ^)^ others have reported lesser skin dose as compared to conventional techniques.^(^ ^6^ ^,^ ^7^ ^)^ But most of the studies do not address the comparison of TPS calculated and measured doses. Chung et al.^(^ ^7^ ^)^ reported large discrepancies in measured dose and dose calculated by commercially available TPS (Pinnacle and Corvus) algorithms. The accurate modeling of dose in the buildup region largely depends on the dose computation algorithm.

In an attempt to improve the accuracy of dose calculation in tissue interface or inhomogeneous region, Varian Medical System (Palo Alto, CA) released a new photon dose calculation algorithm known as anisotropic analytical algorithm (AAA).^(^ ^10^ ^–^ ^11^ ^,^ ^12^ ^,^ ^13^ ^)^ This algorithm uses triple‐source modeling for accurate dose calculation at a point whereby it superimposes the doses from photons of both primary component and secondary scatter photon, and from electron contamination originating from flattening filter, collimator jaws, and accessories. The phase space (particle fluence, energy) parameters are modeled using a Monte Carlo simulation‐derived multiple source model. This consists of a point source for radiation from the primary target, a finite source for extra focal radiation, and a third source to model the electron contamination. It then produces the final dose by superposition and convolution algorithm from these factors. For blocks, beam modifying device and physical wedges, the primary fluence is modified by means of the user‐defined transmission factor. Parameters used to characterize the multileaf collimation (MLC) are the leaf transmission factor and the dosimetric leaf separation. The latter provides the effective dosimetric opening between mechanically closed leaf pairs due to rounded leaf tips.^(^ ^10^ ^–^ ^13^ ^,^ ^14^ ^)^ While very limited studies^(^ ^7^ ^–^ ^15^ ^,^ ^16^ ^)^ have reported comparison of TPS calculated and measured skin dose in clinical treatment conditions, AAA algorithm has not been tested so far to check its reliability and efficiency in the dose calculation in the dose buildup region. In this study, the accuracy of AAA and PBC algorithms available in Eclipse TPS was extensively investigated in non‐clinical as well as clinical treatment conditions for the IMRT dose calculation in both high‐dose buildup and low‐dose buildup regions.

## II. MATERIALS AND METHODS

A commercially available treatment planning system, Eclipse (V 8.6) (Varian Medical System, Palo Alto, CA), was configured for photon pencil beam convolution (PBC) and AAA algorithm using 6 MV X‐rays from Clinac DHX linear accelerator (Varian Medical System, Palo Alto, CA) following manufacturer recommended guidelines and protocols.^(^ ^10^ ^)^ Beam profiles and depth dose curves were measured in a water phantom of RFA 300 Plus with OmniPro Accept software (Wellhofer Scanditronix, Germany) in slow speed and high precision of 0.5 mm stepping mode at five different depths for a number of square field sizes ranging from 2×2 to $40\times 40{\text{cm}}^{2}$. The five different depths for beam profile measurement were at $d_{\max}$ (depth of dose maximum), 5, 10, 20, and 30 cm. This data of beam profiles and depth dose curves for beam configuration were measured using CC13 ion chambers (Wellhofer Scanditronix, Germany). An output factor table at 5 cm depth for a series of rectangular field sizes (X and Y ranging from 1 to 40 cm) was also measured using the same ion chamber. These basic beam data measurements were performed at source to skin distance (SSD) = 100 cm. Commissioning and quality assurance for TPS were performed according to International Atomic Energy Agency (IAEA) Technical Report Series (TRS) report number 430^(^ ^17^ ^)^ and the recommended guidelines and protocols of Varian Linear accelerator.^(^ ^10^ ^)^ The Eclipse TPS and Clinac DHX linear accelerator (which is equipped with 40 pairs of multileaf collimator (MLC) each projecting a leaf width of 1 cm at isocenter) were investigated for accurate modeling of dose distribution in the buildup region in clinical IMRT treatment conditions.

### A. Fabrication of head and neck phantom and treatment planning

**Figure 1:**
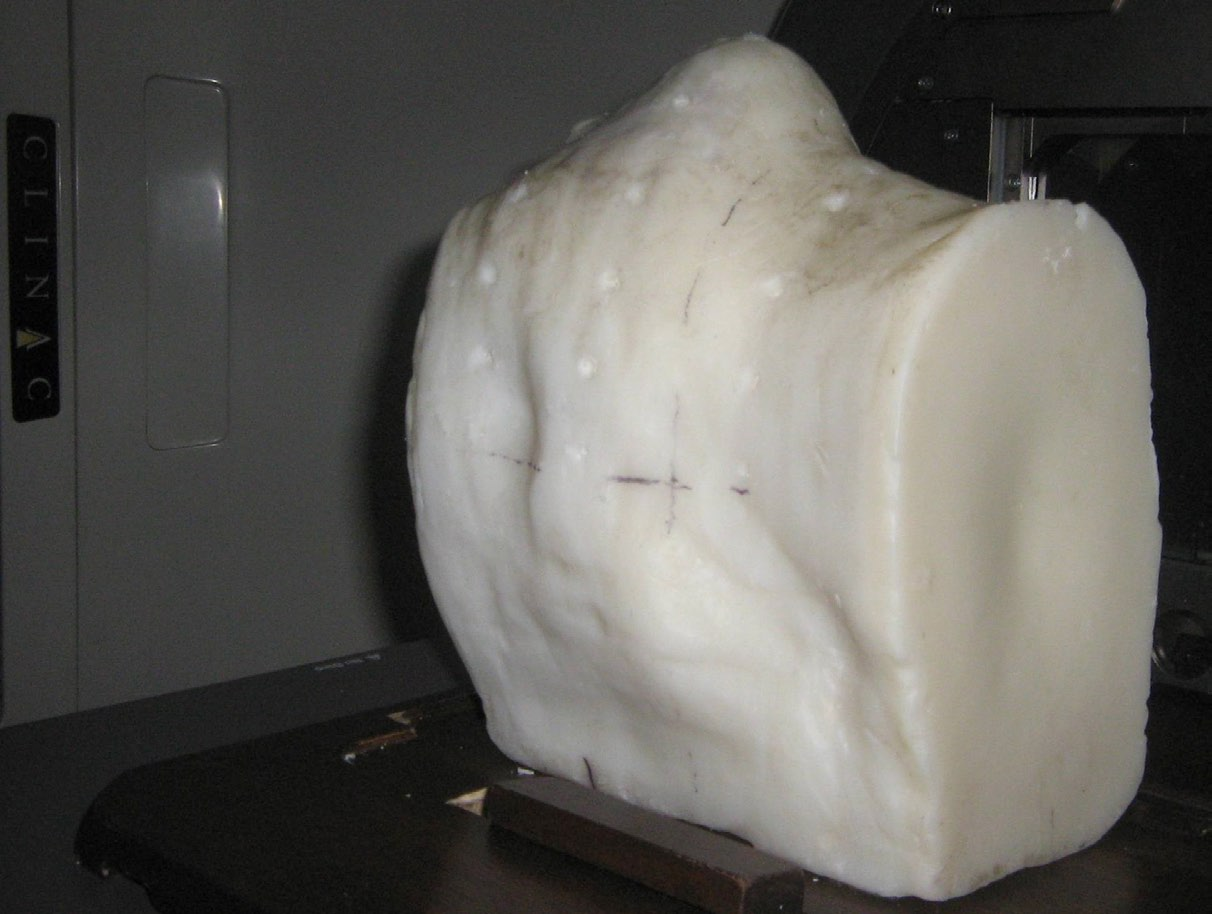
The head and neck wax phantom with registration points for TLD placement (holes of different depths: 2 mm, 4 mm and 6 mm perpendicular to the phantom surface and on the transverse axial positions of the phantom).

An acrylic cast of head and neck region was prepared using VISCO VF Perspex molder (VISCO Enterprise, Mumbai, India) from a patient undergoing IMRT treatment of oropharynx. This cast was prepared exactly in the same condition as the thermoplastic immobilization device that was made for actual treatment planning simulation of the same patient. A head and neck phantom (Fig. 1) was fabricated from paraffin wax using this acrylic cast and carbon fiber base plate, so as to replicate the actual patient and treatment geometry as closely as possible. A thermoplastic mask of this paraffin wax phantom was then prepared under the same condition.

**Figure 2:**
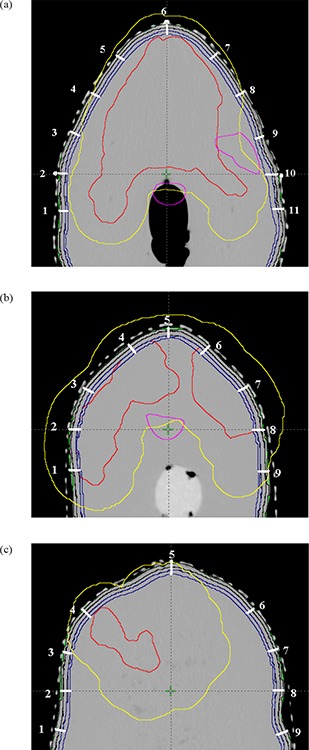
The organs contoured on the CT slice images at isocentre, 5 cm inferior and superior to isocenter, with registration points for TLD placements: spinal cord (magenta color), the PTV to be delivered with 66 Gy (red). The magenta color contour to the right side of the CT axial slice represents the contralateral parotid (left parotid) to be saved; dark blue contours represent three strips of 2 mm thickness at three different depths of 2 mm, 4 mm and 6 mm from the skin of the phantom; yellow contour represents the region of interest which is defined by 1.4 cm extra margin from PTV for the defining of points of high‐ and low‐dose buildup regions.

CT images of this wax head and neck phantom immobilized in the treatment position were acquired at 0.25 cm slices thickness on VFX‐16 multislice CT scanner (GE Medical Systems, San Francisco, CA). A body contour was generated with ‐550 HU (Hounsfield Unit) to exclude the orfit cast from the phantom. Contours containing clinical target volume (CTV) of the actual patient were copied onto the CT datasets of this phantom on Eclipse TPS, and expanded 0.5 cm isotropically to make the planning target volume (PTV). An arbitrary volume called high‐dose buildup region (PTV+1.4cm) was defined by growing a uniform margin of 1.4 cm around PTV (Fig. 2) and will be used for subsequent evaluation of dosimetric outcome from different plans and measurements. All points falling outside this region are considered as the low‐dose region in this study. Similarly, critical organs such as spinal cord, brain stem, larynx and the contra‐lateral parotid gland of the patient were also copied to the phantom. In the TPS, three shells each of 0.2 cm thick were defined at the depths of 0.2 cm, 0.4 cm and 0.6 cm, respectively, from external body surface to quantify the dose in the dose buildup region (Fig. 2). An IMRT plan was created for this phantom on Eclipse treatment planning system (Varian Medical Systems, Palo Alto, CA) using 6 MV X‐rays and seven equally distributed gantry angles. IMRT optimization was done with Helios IMRT optimization software (DVO 8.6, Varian Medical Systems, Palo Alto, CA). Dose optimization constraints assigned for PTV were 66 Gy as lower dose limits to 100% volume and 68 Gy as upper dose limits to 5% volume, to achieve the dose uniformity within the range of 95% and 107% of the prescribed dose 66 Gy to PTV, in accordance with International Commission of Radiation Unit Report (ICRU 50).^(^ ^18^ ^)^ Similarly, the upper dose limits of 48 Gy to 0% volume for spinal cord and 50 Gy to 0% volume for brainstem were set as the dose constraints in dose optimization to achieve the dose within tolerance limits of normal tissue.^(^ ^19^ ^)^ For the contralateral parotid, the upper dose limits were 24 Gy and 20 Gy to the respective 30% and 50% volume. Using the optimal fluence generated by Helios optimization software, two separate patient‐specific IMRT verification plans were created. In one plan, 3D dose were calculated using AAA (version 8.6)^(^ ^10^ ^)^ algorithm while, in the other plan, PBC (version 8.6)^(^ ^14^ ^)^ algorithm was used. A calculation grid size of 0.125 cm was used in both plans.

### B. Dose measurements and verifications

To evaluate the skin (buildup) dose at different locations, three axial planes corresponding to isocenter plane, 5 cm superior and 5 cm inferior to isocentre plane of the head and neck phantom were chosen. Multiple representative points were identified at each plane and at the depth of 0.2, 0.4 and 0.6 cm, respectively. These specific points were defined physically on the phantom by drilling narrow holes perpendicular to the phantom surface. The width of the holes was just sufficient to insert the dosimeter up to a maximum depth of 0.6 cm. These points were localized in the Eclipse TPS, and corresponding doses were calculated using various tools available in the TPS.

**Figure 3:**
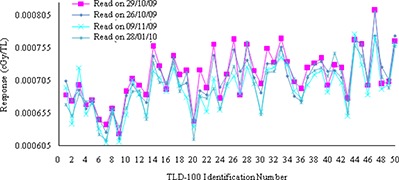
Sensitivity curves against TL chips identification number, generated by reading the TL output on four different dates (26th October 2009, 29th October 2009, 9th November 2009 and 28th January 2010) using UL 300 TLD reader.

**Figure 4:**
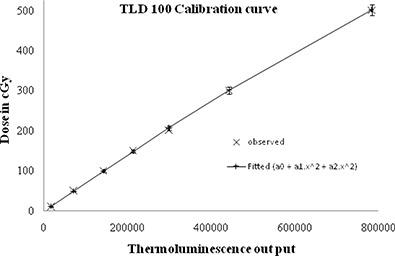
Calibration curve of TLD 100 dosimeters.

Verification of TPS calculated dose in the buildup region was performed using thermoluminescence dosimeter (TLD). TLD‐100 chips (LiF: Mg,TI, Rexon TLD Systems Inc, Beachwood, OH) having dimensions of 0.32cm×0.32cm×0.09cm, were placed at each measurement position corresponding to deeper shell at 0.6 cm. In order to preserve their cleanliness and integrity, these TLD chips were kept in small polyethylene bags. The hollow space above the TLD chips was filled with paraffin wax at the same level of the skin to produce the dose buildup effect on these TLDs. After proper alignment of planned isocenter with the machine isocenter, IMRT plan was delivered on the phantom. This procedure was repeated separately with TLDs distributed on all predefined points at shells located at 0.4 cm and 0.2 cm depths, respectively. Thus, three separate measurements were performed for the same plan without orfit cast. Similarly, another three separate measurements were performed with orfit cast, to evaluate the dose buildup effect of the orfit cast. TL chips used in this study could detect doses ranging from 0.005 Gy to 10 Gy, and 50 TLD chips were preselected from the same batch having reproducibility within ±5% (SD) in the select dose region. These TL chips were assigned a permanent individual identification number. The sensitivity (Fig. 3) of each chip was determined to apply the respective correction factor (correctionfactorz=averagesensitivity/sensitivityofeachTLchip), using a lookup function in Microsoft Excel (as reported in Wagner et al.^(^ ^20^ ^)^). Two TLD chips of ±1% reproducibility and ±1% variation from the average sensitivity were used as control to apply correction factor for every reading cycle. The exposed TL chips were read using a commercially available TLD reader (REXON Model UL‐300, Rexon TLD Systems Inc., Beachwood, OH). Among these 50 TL chips, 14 TL chips of ±1% reproducibility and ±1% variation from the average sensitivity were used for the calibration of this TLD reader using the heat treatment method reported by Yu et al.^(^ ^21^ ^)^ and Meigooni et al.^(^ ^22^ ^)^. A dose calibration curve (Fig. 4) within a range from 0.1 to 5 Gy was generated for the determination of absorbed dose in water phantom. Before the radiation exposure, these TLDs were annealed in an oven at 400°C for 1 hr and a low temperature of 105°C heating for 2 hrs afterward. A pre‐readout annealing of the exposed TL chips was done at 105°C for 15 min and then the dose were read subsequently. Dose measurement reproducibility of the dosimeters was verified within ±2.8% in solid water phantom (RW3). The dose measurement using these TLDs at the representative points in head and neck phantom were compared with the corresponding TPS calculated doses.

## III. RESULTS

**Figure 5:**
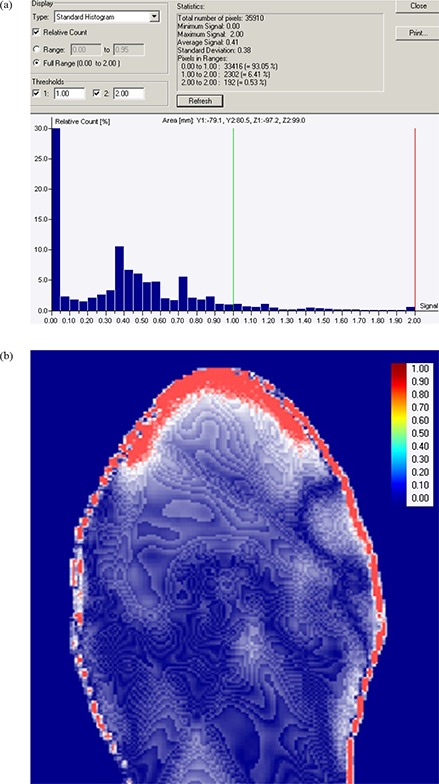
The histogram (a) of gamma values (gamma evaluation parameters of 3% dose difference and 3 mm distance to dose agreement) between AAA and PBC on transverse plane at isocentre; (b) the increase of gamma values from blue color to red color showing the increase in dose difference in dose buildup region.

The dose distribution resulting from two separate plans calculated using PBC and AAA algorithms were compared using gamma values^(^ ^23^ ^)^ in OmniPro IMRT software (Scanditronix Wellhofer, Germany). Gamma acceptance criteria were set as 3% dose difference and 0.3 cm distance to dose agreement (DTA) tolerances. These dose distribution comparisons were evaluated for three representative transverse planes at isocentre, 5 cm superior and 5 cm inferior to isocentre. Figure 5(a) shows the relative histogram of gamma values within the range from 0 to 2.00 on the transverse plane at isocentre. The average gamma values and standard deviation were found as 0.41 and 0.38, respectively, within a region of interest which encompassed the body contour. The percentage of pixel population falling within the gamma acceptance criteria (from 0 to 1.00) and beyond (>1.00) were found to be 97.79% and 2.15%, respectively. An increase of gamma values towards the skin of this phantom, represented by the dense red area in Fig. 5(b), reveals significant dose variation between PBC and AAA algorithms calculations in the high‐dose buildup region proximal to PTV.

**Figure 6:**
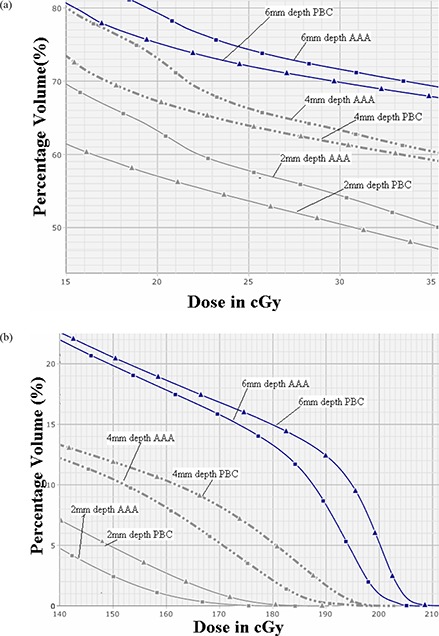
The DVH data (a) of 2 mm strips structures at three different depths of 2 mm (light black continuous lines), 4 mm (light black broken lines) and 6 mm (dark blue continuous lines) for PBC (triangle markers) and AAA (square markers) for low‐dose buildup region (far away from planning target volume, PTV), showing larger dose calculation of AAA over PBC; (b) the same DVH data for high‐dose buildup region (proximal to PTV), showing larger dose calculation by PBC over AAA.

Figures 6(a) and 6(b) show the difference in dose volume histograms of 0.2 cm strips at different depths (0.2 cm, 0.4 cm and 0.6 cm) from the skin calculated using PBC and AAA algorithms of 2.5 mm calculation grid size in low‐dose buildup region and high‐dose buildup regions. At all depths, AAA calculated higher dose than that of PBC in low‐dose buildup region, while in high‐dose buildup region, AAA doses were found to be lower than those of PBC. The results of surface doses on these 0.2 cm strips calculated using both algorithms are also summarized in Table 1. For the surface areas of 1, 50 and $100\;{\text{cm}}^{2}$, PBC overestimated doses as compared to AAA calculated value in the range of 1.34%–3.62% at 0.6 cm depth, 1.74%–2.96% at 0.4 cm depth, and 1.96%–4.06% at 0.2 cm depth, respectively.

**Table 1:** Comparison of the doses on 2 mm strip surfaces at different depths from the skin, calculated by AAA and PBC.

*Depths of 2 mm strips from skins*	*6 mm*		*4 mm*		*2 mm*	
*Algorithm*	*AAA*	*PBC*	*AAA*	*PBC*	*AAA*	*PBC*
Max Dose (Gy)	68.38	71.16	65.43	67.06	61.03	62.98
1 sq cm Dose (Gy)	67.45	68.77	64.34	65.46	57.97	60.07
50 sq cm Dose(Gy)	65.05	66.35	59.93	61.58	50.97	52.34
100 sq cm Dose(Gy)	63.59	65.12	57.19	58.88	47.17	47.80

**Figure 7:**
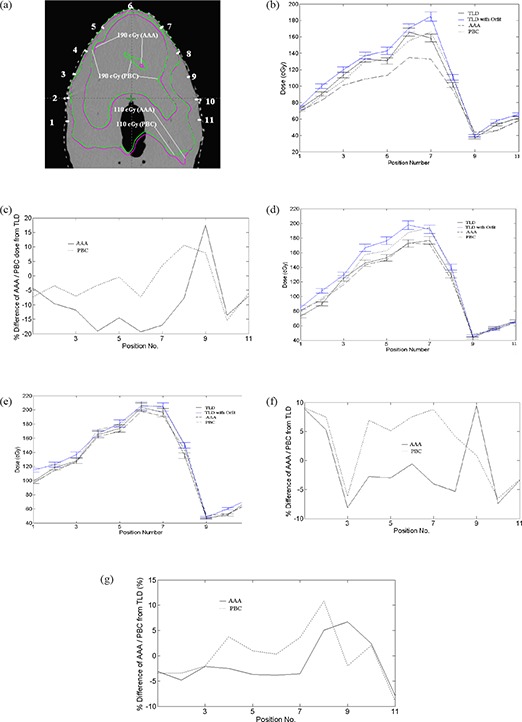
The positions (a) (represented by white spots for TLD placement) and the comparison of dose distributions of PBC and AAA on the transverse axial slices at isocentre, with inner green and magenta isodose curves representing the 190 cGy isodose curves calculated by PBC and AAA respectively, and the outer green and magenta curves representing 110 cGy isodose curves calculated by PBC and AAA respectively. This shows the better homogeneous dose calculated by AAA than that of PBC. Figs. (b), (d) and (e) show the graphs of TLD without orfit (black line=3% error bar in dose), TLD with orfit (blue line=3% error bar in dose), PBC (black short discontinuous line), and AAA (black long discontinuous line) at 2 mm, 4 mm and 6 mm depths, respectively. Figs. (c), (f) and (g) show the graphs of the variation of AAA (black line) and PBC (black discontinuous line) from TLD doses at 2 mm, 4 mm and 6 mm depths, respectively.

Figure 7(a) represents composite dose distributions calculated using AAA and PBC algorithms on the isocentre axial plane. The comparison of doses at the 11 representative points calculated using PBC and AAA and corresponding TLD measured doses are shown in Figs. 7(b), 7(d) and 7(e) for 0.2, 0.4 and 0.6 cm depths, respectively. Figures 7(c), 7(f) and 7(g) represent the variations of AAA and PBC calculated doses from TLD measured doses on the same points and at the same depths, respectively. In general, when the orfit cast is not used for TLD dose measurement, both AAA and PBC overestimate the TLD measured doses – except for the underestimation by AAA at 0.2 cm depth. This is analyzed with the percentage differences of calculated doses from TLD measured doses, normalized to the TLD measured doses as: 100×(calculateddose‐TLDmeasureddose)/TLDmeasureddose. TLD measured doses show better agreement with AAA calculated doses of 0.53%(SD=5.12%) and 0.18%(SD=5.01%) mean differences than the corresponding PBC calculated doses of 4.27%(SD=6.60%) and 1.94%(SD=5.49%) mean differences at 0.4 cm and 0.6 cm depth, respectively (see Table 2(a)). The variation of dose calculation by AAA and PBC from TLD measured doses decreases with depth from 0.2 cm to 0.6 cm. These variations range from 9.17% to 5.01% in the case of AAA and 7.05% to 5.49% in the case of PBC (Table 2(a)). These two algorithms were significantly different from each other at all depths (Table 2(b)). In high‐dose buildup region (within PTV+1.4cm), doses calculated using PBC algorithm overestimate TLD measured doses, whereas AAA underestimates the TLD measured doses at all depths. The percentage differences of calculated doses using AAA and PBC algorithms from TLD measured doses in high‐dose buildup region at three different depths of 0.2, 0.4 and 0.6 cm from skin surface are shown in Table 3(a). It can be seen that in high‐dose buildup region, AAA calculated the doses with an average difference of ‐7.56%(SD=4.73%) lower than the TLD measured doses at 0.2 cm depth, whereas PBC overestimates the doses as compared to TLD measurement with an average difference of 3.75%(SD=5.70%), which is significantly larger (pvalue=0.000) as compared to AAA calculated doses (Table 3(b)). In other depths of 0.4 and 0.6 cm, AAA doses were in agreement with TLD measured doses with different magnitude. While the average percent difference between AAA calculated dose and corresponding TLD measured dose were as small as ‐0.82%(SD=4.24%) and ‐1.10%(SD=4.14%) for 0.4 and 0.6 cm depth, respectively, the corresponding values from PBC were as large as 5.84%(SD=4.38%) and 2.40%(SD=4.76%), respectively (Table 3(a)). PBC calculated doses were significantly larger than those of AAA with *p* value of 0.001 and 0.005 at 0.4 cm and 0.6 cm depths, respectively (Table 3(b)).

**Table 2(a):** Statistical distribution of percentage dose difference of calculated dose (using AAA and PBC algorithms) from TLD measured dose normalized to TLD measured doses in high‐dose buildup region at all dose measurement points.

		*No Orft*			*With Orft*		
*Depth From Skin*	*Algorithm*	*% mean dose differences from TLD (SD)*	*Minimum*	*Maximum*	*% mean dose differences from TLD (SD)*	*Minimum*	*Maximum*
2 mm	AAA	‐4.71 (9.17)	‐19.32	17.59	‐13.35 (8.46)	‐28.77	9.37
	PBC	2.09 (7.05)	‐15.29	13.10	‐7.24 (5.28)	‐22.14	6.57
4 mm	AA	0.53 (5.12)	‐8.10	11.95	‐8.21 (5.27)	‐15.86	3.76
	PBC	4.27 (6.60)	‐6.12	11.48	‐4.94 (4.08)	‐10.4	5.53
6 mm	AAA	0.18 (5.01)	‐9.12	13.41	‐5.74 (5.28)	‐16.62	7.54
	PBC	1.94 (5.49)	‐9.01	11.08	‐4.01 (6.70)	‐16.88	10.9

**Table 2(b):** Statistical analysis of the percent dose difference of dose calculation in high‐dose buildup region at 2 mm, 4 mm and 6 mm depths from the skin by two algorithms (AAA and PBC) using paired t‐test.

	*No Orft*		*With Orft*	
*Depth From Skin*	*% mean dose differences of AAA from PBC (SD)*	*p‐value*	*% mean dose differences of AAA from PBC (SD)*	*p‐value*
2 mm	‐6.80 (7.67)	0.000	‐6.11 (6.92)	0.000
4 mm	‐3.74 (5.11)	0.000	‐3.27 (4.86)	0.001
6 mm	‐1.76 (3.82)	0.019	‐1.72 (3.53)	0.014

**Table 3(a):** Statistical distribution of percentage dose difference of calculated dose (using AAA and PBC algorithms) from TLD measured dose normalized to TLD measured doses in high‐dose buildup region proximal to PTV (1.4 cm extra margin from PTV).

		*No Orft*			*With Orft*		
*Depth From Skin*	*Algorithm*	*% mean dose differences from TLD (SD)*	*Minimum*	*Maximum*	*% mean dose differences from TLD (SD)*	*Minimum*	*Maximum*
2 mm	AAA	‐7.56 (4.73)	‐19.32	2.21	‐17 (5.31)	‐28.77	‐9.97
	PBC	3.75 (5.70)	‐7.25	13.10	‐6.84 (3.13)	‐11.1	‐1.87
4 mm	AAA	‐0.82 (4.24)	‐8.10	6.65	‐9.85 (4.08)	‐15.86	‐1.78
	PBC	5.84 (4.38)	‐6.12	11.48	‐3.81 (3.81)	‐9.16	2.68
6 mm	AAA	‐1.10 (4.14)	‐9.12	8.24	‐4.91 (3.94)	‐12.7	4.03
	PBC	2.40 (4.63)	‐6.56	11.08	‐1.54 (4.44)	‐10.2	6.76

SD = standard deviation which includes the uncertainties of 2.8 % and 0.9 % due to TLD dosimetry and directional dependence over the dosimetry data variation of this study using quadrature sum.

Negative sign (‐) indicates the calculated values were lesser than the TLD measured values; positive numbers indicate the calculated values were higher than the TLD measured values.

**Table 3(b):** Statistical analysis of the percent dose difference of dose calculation in high‐dose buildup region proximal to PTV (1.4 cm extra margin from PTV) at specific points on 2 mm strips surface at different depths from the skin by two algorithms (AAA and PBC) using Wilcoxon signed‐rank test showing significant differences between these two at all depths.

*(PBC‐meas)/meas vs (AAA‐meas)/meas*	*Type of Ranks*	*N*	*Mean Rank*	*Sum of Ranks*	*Z*	*p‐value (2‐tail)*
	Negative Ranks	0	0.00	0.00	‐3.29	0.001
2 mm Depth	Positive Ranks	14	7.50	105.00
	Ties	0
	Total	14
	Negative Ranks	0	0.00	0.00	‐3.52	0.000
4 mm Depth	Positive Ranks	16	8.50	136.00
	Ties	0
	Total	16
	Negative Ranks	0	0.00	0.00	‐3.62	0.000
6 mm Depth	Positive Ranks	17	9.00	153.00
	Ties	0
	Total	17

In contrast to previous findings of high‐dose buildup region, both AAA and PBC overestimated the doses as compared to TLD measured doses at all depths in low‐dose buildup region (beyond PTV+1.4cm), except for the dose underestimation by AAA at 0.2 cm depth (Table 4(a)). However, similar to high‐dose buildup region, the variation of AAA calculated and TLD measured dose was smaller as compared to the corresponding values from PBC calculation. In Figs. 7(b), 7(d) and 7(e) of low‐dose buildup regions, both AAA and PBC also overestimate the TLD measured doses, except the dose underestimation by AAA at 0.2 cm depth. The variations of PBC and AAA calculated doses from those of TLD measured were largest on the points in low‐dose buildup region (Table 4(a) and Figs. 7(c), 7(f) and 7 (g)). The average percent differences between AAA calculated doses and corresponding TLD measured doses at 0.2, 0.4 and 0.6 cm depth were respectively ‐2.05%(SD=10.21%),2.82%(SD=5.38%) and 2.00%(SD=5.73%); those of PBC calculated doses from TLD measured doses were 0.55%(SD=8.0%),2.99%(SD=8.47%) and 1.29%(SD=6.69%) at 0.2, 0.4 and 0.6 cm depths, respectively (Table 4(a)). The differences between AAA and PBC calculated doses in this low‐dose buildup region at all depths were not statistically significant (Table 4(b)). This result does not match with those of Fig. 6(a), in which the comparison of these two algorithms was done at relatively lower dose level. When the orfit is used for TLD dose measurements, there was increase in the dose buildup effect, as shown in Tables 2(a), 3(a) and 4(a). The mean difference of doses calculated by both algorithms from TLD measured doses were reduced from ‐13.35%(SD=0.846%) to ‐5.74%(SD=5.28%) for AAA, and ‐7.24%(SD=5.28%) to ‐4.01% (6.70%) for PBC with depth (from 0.2 cm to 0.6 cm) (Table 2(a)).

**Table 4(a):** Statistical distribution of percentage dose difference of calculated dose (using AAA and PBC algorithms) from TLD measured dose normalized to TLD measured doses in low‐dose buildup region far away from PTV (beyond 1.4 cm extra margin from PTV).

		*No Orft*			*With Orft*		
*Depth From Skin*	*Algorithm*	*% mean dose differences from TLD (SD)*	*Minimum*	*Maximum*	*% mean dose differences from TLD (SD)*	*Minimum*	*Maximum*
2 mm	AAA	‐2.05 (10.21)	‐13.61	17.59	‐9.95 (9.56)	‐20.59	9.37
	PBC	0.55 (8.0)	‐15.29	11.95	‐7.62 (6.81)	‐22.14	6.57
4 mm	AAA	2.82 (5.38)	‐7.39	11.95	‐3.9 (4.52)	‐12.25	3.76
	PBC	2.99 (8.47)	‐6.12	9.47	‐3.93 (4.82)	‐10.4	5.53
6 mm	AAA	2.00 (5.73)	‐7.94	13.41	‐6.04 (7.11)	‐16.62	7.54
	PBC	1.29 (6.69)	‐9.01	16.96	‐6.64 (8.42)	‐16.88	10.9

SD = standard deviation which includes the uncertainties of 2.8 % and 0.9 % due to TLD dosimetry and directional dependence over the dosimetry data variation of this study using quadrature sum.

Negative sign (‐) indicates the calculated values were lesser than the TLD measured values; positive numbers indicate the calculated values were higher than the TLD measured values.

**Table 4(b):** Statistical analysis of the percent dose difference of dose calculation in low‐dose buildup region far away from PTV (beyond 1.4 cm extra margin from PTV) at specific points on 2 mm strips surface at different depth from skin by two algorithms (AAA and PBC) using Wilcoxon signed‐tank test showing significant differences between these two at all depths.

*(PBC‐meas)/meas vs (AAA‐meas)/meas*	*Type of Ranks*	*N*	*Mean Rank*	*Sum of Ranks*	*Z*	*p‐value (2‐tail)*
	Negative Ranks	5	7.60	38.00	‐1.25	0.211
2 mm Depth	Positive Ranks	10	8.20	82.00
	Ties	0
	Total	15
	Negative Ranks	4	8.55	34.00	‐0.805	0.422
4 mm Depth	Positive Ranks	9	6.33	57.00
	Ties	0
	Total	13
	Negative Ranks	7	5.43	38.00	‐0.078	0.937
6 mm Depth	Positive Ranks	5	8.00	40.00
	Ties	0
	Total	12

## IV. DISCUSSION

**Figure 8:**
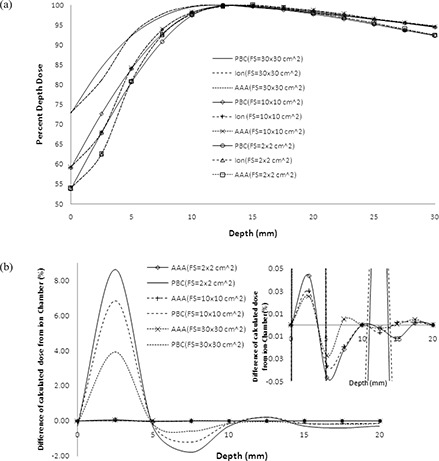
Comparison (a) of calculated percent depth dose (PDD) of AAA (shorter discontinuous lines), PBC (continuous lines) and measured PDD (longer discontinuous lines) using ion chamber for field sizes (FS) of $2\times 2{\text{cm}}^{2}$ (no marker), $10\times 10{\text{cm}}^{2}$ (marker) and $30\times 30{\text{cm}}^{2}$ (marker). The difference of PDD (b), calculated by PBC (no marker) and AAA (marker) from those of ion chamber measured for field sizes of $2\times 2{\text{cm}}^{2}$ (continuous lines), $10\times 10{\text{cm}}^{2}$ (longer discontinuous lines) and $30\times 30{\text{cm}}^{2}$ (shorter discontinuous lines). The inset the enlarged PDD differences in the scale within ±0.05%.

**Figure 9:**
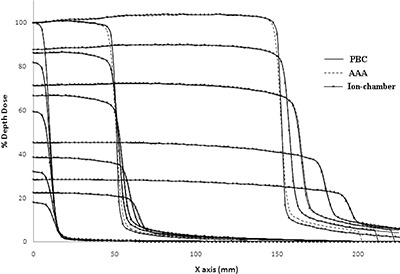
Comparison of calculated dose profiles (AAA and PBC) at depth of dose maximum $\left(d_{\max}\right)$, 5 cm, 10 cm, 20 cm and 30 cm depths and measured dose profiles using CC13 ion chamber at the same depths for field sizes of $2\times 2{\text{cm}}^{2},10\times 10{\text{cm}}^{2}$ and $30\times 30{\text{cm}}^{2}$.

**Figure 10:**
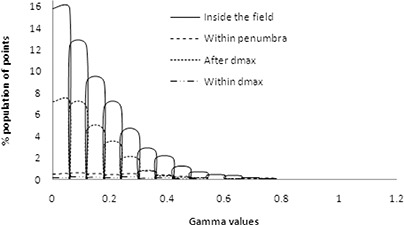
Gamma values calculated which pass the tolerance dose of 3% and distance to dose agreement of 1 mm.

Our beam data configuration showed that the basic depth dose beam data were reproduced by the AAA algorithm within 0.05% in the dose buildup region and 0.02% beyond the depth of dose maximum $\left(d_{\max}\right)$, whereas PBC calculated dose showed overall maximum deviation of 8.61% (field size of $2\times 2{\text{cm}}^{2}$) from the basic beam data in the dose buildup region and ‐0.48% beyond $d_{\max}$ (Figs. 8(a) and 8(b)). Figure 9 shows the comparison of AAA with the PBC calculated profiles from ion chamber measured dose profiles. The reproducibility of AAA calculated depth doses and dose profiles were evaluated by the histogram of gamma values which passed the tolerance limits of relative dose difference of 3% and distance to dose agreement of 1 mm. These gamma values were found to be in 100% agreement within the depth of dose maximum, 100% after depth of dose maximum, 99.98% inside the profiles and 100% in the penumbra region (Fig. 10). All these gamma values were more than 99.0%, which is the acceptance criteria of commissioning of AAA algorithm in Eclipse TPS as reported in Eclipse algorithm reference guide lines of Varian Medical Systems.^(^ ^10^ ^)^ Van Esch et al.^(^ ^13^ ^)^ also reported that the optimization procedure for the configuration of algorithm was successful in reproducing the basic beam data with an overall accuracy of 3%, 1 mm in the dose buildup region and 1%, 1 mm elsewhere. The results of our study match well with those of Chung et al.,^(^ ^7^ ^)^ which showed the dose overestimation by two TPS algorithms. Our TLD measurements in all dose measurement points in the dose buildup region showed dose overestimation by both AAA and PBC algorithms, except the dose underestimation by AAA at 2 mm depth. The dose underestimation was the average difference of ‐4.71%(SD=9.17%,maximum=17.59% and minimum=‐19.32%) whereas the dose overestimation by PBC algorithms was an average dose difference of 2.09%(SD=7.05%,maximum=13.10% and minimum=‐15.29%) on 0.2 cm strips at 0.2 cm depth (Table 2(a)). Significant differences between these two algorithms were observed in the dose calculation at 0.2 cm depth from the skin in this region with *p* value of 0.000 (Table 2(b)). At 0.4 cm and 0.6 cm depths, AAA showed significant improvement of dose calculation and was found closer to TLD doses, with the mean difference from TLD doses as 0.53%(S=5.12%,maximum=11.95% and minimum=‐8.10%) and 0.18%(SD=5.01%,maximum=13.41% and

Minimum=‐9.12%), respectively, as compared with that of 4.27%(SD=6.60%,maximum=11.48% and minimum=‐6.12%) and 1.94%(SD=5.49%,maximum=11.08% and minimum=‐9.01%) in the case of PBC at their respective depths. The large variations, represented by standard deviations of AAA (SD=9.17%) and PBC (SD=7.05%) calculated doses from TLD measured doses at 0.2 cm reveal that both AAA and PBC algorithms still have limitations in dose calculation at 0.2 cm depth. The larger mean difference of 4.27% and standard deviation of 6.6% indicates that there is limitation of dose calculation by PBC at 0.4 cm depth. This may be attributed to the fact that AAA calculation takes into account the consideration of electron densities of local neighboring points in 16 lateral directions^(^ ^10^ ^,^ ^24^ ^)^ using the scattering kernels scaled along the depths and lateral directions,^(^ ^25^ ^)^ which takes into account the contour irregularities. This results in the gradual change of dose gradient in the regions closer to skin contour as well as at a depth, and thus improves the dose uniformity (Figs. 6(b) and 7(a)). The improvement of dose calculations in this high dose buildup occurred due to the utilization of electron contamination source and second source for photon in optimization method for beam configuration. The exit beam also contributes a significant dose on these 0.2 cm strips. In low‐dose buildup region, both AAA and PBC overestimated the TLD measured doses, and AAA doses were found significantly higher than those of PBC. This is possibly occurred due to utilization of electron contamination source in optimization method for beam configuration of AAA algorithm and inabilities of dose calculation by PBC algorithm in this region. There was also an increase in the dose nonuniformity (Figs. 6(b) and 7(a)) as well as the dose maximum with PBC calculation as compared with that of AAA algorithm. This may be due to the uses of single scattering kernel and effective path length of modified Batho power law in PBC algorithm. Davidson et al.^(^ ^26^ ^)^ also reported that the use of the pencil‐beam algorithm with only an effective path length correction may result in the dose to the target being overestimated.

In a clinical treatment condition, the use of orfit cast increases the dose buildup effect and this buildup effect decreases with depth. The present dose calculations using AAA and PBC algorithms did not consider the X‐ray attenuations through the base plate and carbon fiber table top. Such attenuations lead to the maximum dose attenuation of 15%, as reported by Vieira et al.^(^ ^27^ ^)^ This TLD dosimetry does not take into account the effective point of measurement, which is not possible under clinical treatment conditions. These might be reasons why there were large variations between calculated and measured dose especially at 2 mm. These uncertainties lead us to perform statistical analysis to evaluate the efficiency of these two algorithms with the following conclusion.

## V. CONCLUSIONS

In the seven‐field IMRT plan, doses calculated using PBC algorithm were higher than those from AAA algorithm in high‐dose buildup region and were lower in low‐dose buildup region. AAA algorithm significantly improved the dose calculations at buildup region (0.4 cm to 0.6 cm), especially in high‐dose buildup region (proximal to PTV) and was found to be closer to TLD measured doses as compared to PBC algorithm. PBC was overestimating the doses from TLD measured doses in this high‐dose buildup region. Again, both AAA and PBC doses overestimate the TLD measured doses in low‐dose buildup region, except at 0.2 cm depth, and AAA doses were closer to TLD measured doses. The present study concludes that there is a limitation of dose calculation by both algorithms within 0.2 cm depth in the dose buildup region proximal to PTV as well as farther away from PTV. However, AAA algorithm is found to be more reliable on the points at all depths in high‐dose and low‐dose buildup regions compared with PBC algorithm.
